# Trends in subcutaneous depot medroxyprogesterone acetate (DMPA-SC) use in Burkina Faso, the Democratic Republic of Congo and Uganda

**DOI:** 10.1016/j.conx.2019.100013

**Published:** 2019-11-09

**Authors:** Philip Anglewicz, Pierre Akilimali, Georges Guiella, Patrick Kayembe, Simon P.S. Kibira, Fredrick Makumbi, Amy Tsui, Scott Radloff

**Affiliations:** a Johns Hopkins Bloomberg School of Public Health, Department of Population, Family and Reproductive Health, 615 N. Wolfe Street E4533, Baltimore, MD 21205, USA; b School of Public Health, University of Kinshasa, Kinshasa, Democratic Republic of the Congo; c Institut Supérieur des Sciences de la Population, University of Ouagadougou, Ouagadougou, Burkina Faso; d Department of Community Health and Behavioral Sciences, Makerere University School of Public Health, Kampala, Uganda; e Department of Epidemiology and Biostatistics, Makerere University School of Public Health, Kampala, Uganda

**Keywords:** DMPA-SC, Sayana® Press, Family planning, Contraceptive use, Burkina Faso, Democratic Republic of Congo, Uganda

## Abstract

**Objectives:**

Subcutaneous depot medroxyprogesterone acetate (DMPA-SC) is seen as a valuable innovation in family planning, but little is known about trends in DMPA-SC use or characteristics of users. Using data from Burkina Faso, the Democratic Republic of Congo (DRC) and Uganda, we measured trends in DMPA-SC and identified characteristics associated with DMPA-SC use.

**Study design:**

We used repeated cross-sectional representative data collected between 2016 and 2019. First, we plotted trends in DMPA-SC use for all women and married women. Next, we presented the sociodemographic and family-planning-related characteristics of DMPA-SC users. Finally, we conducted weighted multivariate logistic regression analysis to examine how DMPA-SC users were different from women (1) using all other modern methods combined and (2) not using any modern method.

**Results:**

DMPA-SC use increased monotonically in all three countries. Many DMPA-SC users were first-time users of modern contraception (54.5% in Burkina Faso, 34.6% in DRC, 50.7% in Uganda). Never-married women had lower odds than married women of using DMPA-SC (compared to other modern methods) in all three countries [Burkina Faso adjusted odds ratio (AOR) 0.40, 95% confidence interval (95% CI) 0.20–0.80; DRC AOR 0.31 95% CI 0.10–0.93; Uganda AOR 0.24; 95% CI 0.08–0.71]. Level of education was positively associated with DMPA-SC use (compared to no use) (Burkina Faso AOR 1.79; 95% CI 1.03–3.14; Uganda AOR 3.23; 95% CI 1.33–7.84).

**Conclusions:**

DMPA-SC is a rapidly growing method in these settings. Despite the comparable levels of and increases in use for all three countries, the characteristics associated with DMPA-SC use generally differed across countries.

**Implications:**

This is the first analysis of patterns of DMPA-SC use with representative data for African countries. Our results confirm that DMPA-SC is increasingly popular, although the profile of users varies across settings.

## 1 Introduction

Subcutaneous depot medroxyprogesterone acetate (DMPA-SC), also known by the product name Sayana® Press, is seen as a valuable innovation in family planning. Compared to other contraceptive methods, the benefits of DMPA-SC include ease of use, few side effects, fast administration, less pain and greater effectiveness [[1], [2], [3], [4]].

Studies suggest that DMPA-SC is widely acceptable in sub-Saharan Africa (SSA). Pilot testing in Burkina Faso, Niger, Senegal and Uganda from 2014 to 2016 showed that DMPA-SC can add value to national family planning programs [5]. Some users preferred DMPA-SC over intramuscular depot medroxyprogesterone acetate (DMPA-IM) [1,6]. Since the pilots, some countries, like Burkina Faso, have expanded distribution of DMPA-SC [7] and tested different approaches to distribution, such as self-injection and community-level distribution [5,7,8].

Given that the introduction of DMPA-SC is still in the early stages in many countries, little is known about the characteristics of DMPA-SC users. Who are they, and how are they different from users of other modern methods and women not using modern methods? To what extent do the characteristics of DMPA-SC users vary across settings? Some have speculated that DMPA-SC use may be particularly appealing to young women [2,5,9] and first-time users [5], but this has seldom been examined, primarily due to data limitations. The small body of existing research on the characteristics of DMPA-SC users has either relatively few user characteristics, used small sample sizes without broad geographic representation or both, and has not compared DMPA-SC users with users of other methods or nonusers [3,7,9,10].

In this research, we use representative data from Burkina Faso, the Democratic Republic of Congo (DRC) and Uganda to identify characteristics associated with DMPA-SC use and how these characteristics vary across countries. First, we measure trends in DMPA-SC use over time. Next, we provide characteristics of DMPA-SC users in each country, including the percentage who were first-time users of modern contraception. Finally, we identify characteristics associated with DMPA-SC use compared to all other modern methods combined and nonuse of modern contraception.

## 2 Data

Data for representative analysis of DMPA-SC users are rare. National health information systems are not usually equipped to track DMPA-SC use because they typically group methods by general type (e.g., pill, injectable, implant) and do not distinguish between DMPA-SC and DMPA-IM [5]. They also typically cover only public facilities, which is a limitation in settings where private facilities are important sources of contraceptive services [11]. Common sources of data for contraceptive use, like Demographic and Health Surveys, have not yet specifically measured use of DMPA-SC.

We used data from the Performance Monitoring and Accountability 2020 Project (PMA2020) [12]. Since 2013, PMA2020 has operated in 11 geographies in Africa and Asia. In most of these settings, PMA2020 collected nationally representative data. To do so, PMA2020 used a multistage stratified cluster design to draw a probability sample of households and females of childbearing age. Datasets are made publicly available within 6 months of data collection and can be obtained at www.pma2020.org.

An innovation of PMA2020 is the use of female resident enumerators (REs) recruited from within or near sampled enumeration areas (EAs) and trained to collect data using smartphone technologies. REs map and list every household within the EA to create a sampling frame from which households are randomly selected. The RE completes a household roster for each selected household, and all females age 15 to 49 are asked to be interviewed (for more detail, see [12]). The use of REs is an important innovation as it may yield more accurate data than the typical approach of using interviewers from outside the study setting [13].

Three countries are included in this analysis: Burkina Faso, Uganda and DRC. Data from the first two countries are nationally representative, while data from DRC are representative of the two provinces of Kinshasa and Kongo Central. We selected these three countries because they all have multiple rounds of data in which DMPA-SC use was measured and has increased to non-negligible percentages of use. Other PMA countries were not included in the analysis because they either did not measure DMPA-SC at all or in more than one round (India, Ghana, Indonesia, Ethiopia, Kenya, Ivory Coast), or had a DMPA-SC prevalence of less than 1% among all women (Niger, Nigeria). The specific rounds of PMA data used are from 2016, 2017, 2018 and 2019 for Burkina Faso; 2016, 2017 and 2018 for DRC; and 2017 and 2018 for Uganda.

Ethical approval for conducting PMA2020 was received from institutional review boards (IRBs) in each country (Burkina Faso, Comité d’Ethique Pour La Recherche en Santé, Ministère de l’Enseignement Supérieur, de la Recherche Scientifique et de l’Innovation; Uganda, Makerere University School of Public Health Higher Degrees, Research and Ethics Committee and the Uganda National Council for Science and Technology; and DRC, University of Kinshasa School of Public Health) and Johns Hopkins Bloomberg School of Public Health. All respondents were approached for informed consent before enrollment in the study, and the relevant ethical review boards approved all consent procedures. Because all analyses here were using publicly available data (without any identifying information), IRB approval was not necessary for the analysis in this paper.

### 2.1 Funding

This article was developed under grant #OPP1079004 awarded to Johns Hopkins Bloomberg School of Public Health by the 10.13039/100000865Bill & Melinda Gates Foundation. The funding source was not involved in the design or conduct of the research.

### 2.2 Measures

Current contraceptive use was measured by PMA2020 through the following question: “Are you or your partner currently doing something or using any method to delay or avoid getting pregnant?” If the woman reported using contraception, she was asked to name the method or methods she was using. We define modern contraceptive methods as hormonal and barrier methods, sterilization, emergency contraception, lactational amenorrhea method and the standard days/cycle beads method. If the woman reported using an injectable, she was asked “Was the injection administered via syringe or small needle?”, and was shown an image of both DMPA-SC and DMPA-IM so she could provide an accurate distinction. We identified the percentage of DMPA-SC users whose first method was DMPA-SC (as opposed to switching to DMPA-SC from another method) through the following question: “Which method did you first use to delay or avoid getting pregnant?”

We next focused on characteristics of DMPA-SC users, including sociodemographic measures such as age, number of lifetime births, marital status, level of education, household wealth and urban/rural residence. Household wealth was measured using a constructed index based on ownership of 25 household durable assets, house and roof material, livestock ownership and water source, which was converted into quintiles. We also included measures of family planning programming: whether participants were exposed to a family planning message via radio, television or a magazine in the past 12 months. Finally, we included an additive index of FP method knowledge (ranging from 0 to 13, with 0 meaning respondent does not know any contraceptive methods and 13 being respondent knows 13 modern contraceptive methods).

### 2.3 Analytic methods

We conducted our analysis in several steps, beginning with trends in DMPA-SC use in each country. We plotted the (weighted) percentages of married and all women using DMPA-SC for the rounds of PMA2020 mentioned above in each of the three countries.

We next tabulated characteristics of DMPA-SC users in each country. We described DMPA-SC users by age; number of lifetime births; marital status; level of education; household wealth quintile; exposure to FP messages via radio, television and magazine; urban residence (compared to rural, not available for DRC as rural/urban designation was not included in the sampling frame); number of FP methods known and the percentage for whom DMPA-SC use was their first modern method.

Finally, we conducted multivariate analysis to examine whether and how users of DMPA-SC were different from women (1) using all other modern methods combined and (2) not using modern methods (which included women using traditional methods). To do so, we used logistic regression in which the binary dependent variable was DMPA-SC (with value of “1”) compared to the other two categories above (value of “0”). Independent variables included were age (separated into 5-year age intervals from 15 to 49 years), number of births (divided into categories of 0–1, 2–5, 6 or more), highest level of educational attainment (none, primary, secondary, tertiary), marital status (currently married/living together, divorced/widowed, never married), wealth quintile, the FP program exposure measures, number of contraceptive methods known (0–13) and an indicator for PMA2020 survey year. We presented odds ratios and 95% confidence intervals (CIs) for all regression results. The few missing values were considered missing at random. We accounted for the study design features and nonresponse by using survey weights in our analysis. An alternative approach would be to use a multinomial regression models with DMPA-SC use as the reference category (compared to other modern methods and not using any methods), but we use the logistic regressions described above because this produces coefficients for DMPA-SC use specifically, which is of central interest to this study.

## 3 Results

**Fig. 1:**
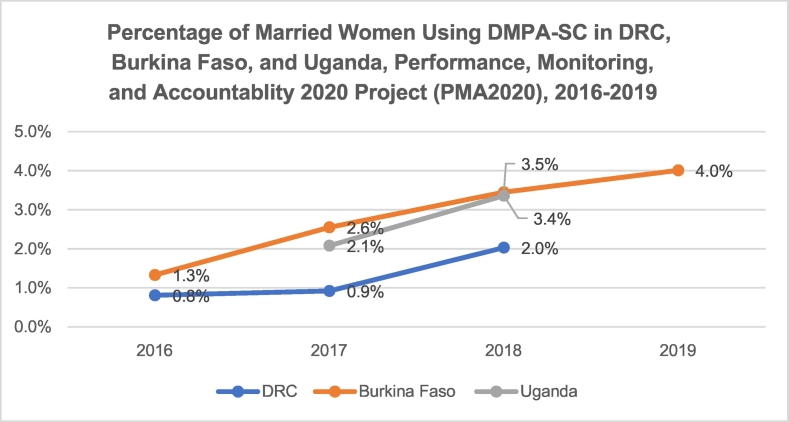
Percentage of married women using DMPA-SC in DRC, Burkina Faso and Uganda: PMA2020, 2016–2019.

**Fig. 2:**
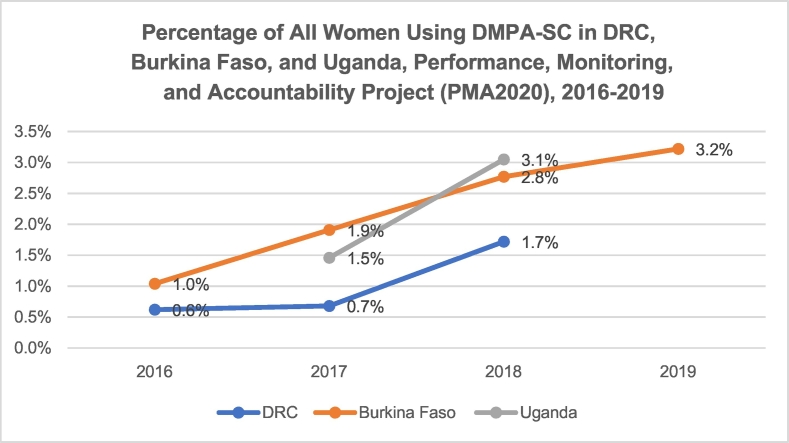
Percentage of all women using DMPA-SC in DRC, Burkina Faso and Uganda: PMA2020, 2016–2019.

Although DMPA-SC was used by only a small percentage of women in our three geographies, use increased monotonically between 2016 and 2019 in DRC, Burkina Faso and Uganda. As shown in Fig. 1, Fig. 2, in Burkina Faso, the percentage of all women using DMPA-SC increased from 1.0% to 3.2% and from 1.3% to 4.0% among married women between 2016 and 2019. Use of DMPA-SC doubled among all women in a 1-year period in Uganda, from 1.5% to 3.1%, and nearly tripled in DRC between 2016 and 2018, from 0.6% to 1.7%.

Table 1 shows characteristics of DMPA-SC users. Some of these characteristics were consistent across geographies: in all three countries, the age groups in the 20s made up the highest percentage of DMPA-SC users (20–24 for DRC, 25–29 for Burkina Faso and Uganda); the majority of DMPA-SC users had three to five children; and the vast majority were currently married. However, there were also some notable differences in user profile across countries: the majority of DMPA-SC users were in the lowest three wealth quintiles in Burkina Faso but were in the highest three quintiles in DRC and Uganda. There were also differences by education; most DMPA-SC users in Burkina Faso had no education, while the largest category of users in Uganda and DRC was composed of women with at least some secondary school education.

**Table 1:** Characteristics of DMPA-SC users in Burkina Faso, DRC and Uganda:PMA2020, 2016–2018

*N* =	Burkina Faso	DRC	Uganda
	293	109	168
*Age category*			
15–19	7.5%	5.3%	5.2%
20–24	23.4%	28.9%	22.2%
25–29	28.1%	19.2%	31.0%
30–34	18.7%	15.9%	18.3%
35–39	13.3%	13.1%	14.5%
40–44	6.6%	9.3%	5.7%
45–49	2.3%	8.2%	3.2%

*Number of children*			
0–1	20.5%	35.9%	24.2%
2–5	58.3%	50.0%	55.0%
6 +	21.2%	14.1%	20.7%

*Education*			
None	63.0%	3.5%	4.9%
Primary	20.9%	26.9%	40.7%
Secondary	15.3%	69.1%	49.4%
Tertiary	0.8%	0.6%	4.9%

*Marital status*			
Married	93.4%	71.3%	86.0%
Divorced/widowed	2.3%	12.7%	9.7%
Never married	4.3%	16.1%	4.3%

*Wealth quintile*			
1 (lowest)	17.8%	5.6%	17.7%
2	26.4%	12.5%	17.2%
3	24.9%	14.4%	21.1%
4	17.7%	24.5%	26.8%
5	13.2%	43.0%	17.3%
Heard FP on radio	68.5%	46.9%	76.3%
Saw FP on TV	36.6%	38.3%	30.8%
Saw FP in magazine	12.6%	8.7%	17.0%
Mean number of FP methods known	3.9	5.1	9.0
Urban residence	19.3%	----	20.2%
First method was DMPA-SC	54.5%	34.6%	50.7%

Many users of DMPA-SC were first-time users of contraception. In Burkina Faso and Uganda, DMPA-SC was the first contraceptive method for most users (at 54.5% and 50.7%, respectively). A smaller but not insubstantial 34.6% of DMPA-SC users in DRC were first-time users.

In our first set of multivariate results, we compared characteristics of DMPA-SC users with users of all other modern methods combined (Table 2). One result was consistent across countries: never married women had significantly lower odds of using DMPA-SC compared to other modern methods in all three countries. There was an increase in DMPA-SC use over time compared to other modern methods in Burkina Faso and Uganda. Other characteristics differ across settings: wealth is associated with DMPA-SC use only in DRC, where the highest wealth quintile was associated with greater odds of DMPA-SC use [adjusted odds ratios (AORs) 4.82, 95% CI 1.50–15.47]. Other differences were that DMPA-SC users were more likely to be 20–24 compared to 15–19 (AOR 2.68, 95% CI 1.13–6.38) and more likely to have heard about FP on the radio (AOR 1.79, 95% CI 1.09–2.94) in DRC, and less likely to have two to five2-5 children compared to zero to 1 child in Uganda.

**Table 2:** Weighted logistic regression results for differences in characteristics between DMPA-SC and all other users of modern methods combined, Burkina Faso, DRC and Uganda: PMA2020, 2016–2019

*N* =	Burkina Faso			DRC			Uganda		
	AOR	95% CI		AOR	95% CI		AOR	95% CI	
	3533			2791			2280		
*Age category*									
15–19 (ref)	**----**	**----**	**----**	**----**	**----**	**----**	**----**	**----**	**----**
20–24	1.07	0.61	1.88	**2.68**	**1.13**	**6.38**	1.62	0.73	3.63
25–29	0.93	0.46	1.91	2.02	0.71	5.77	2.20	0.98	4.94
30–34	0.65	0.27	1.57	1.92	0.59	6.25	1.72	0.74	3.99
35–39	0.70	0.30	1.64	1.73	0.56	5.33	1.68	0.64	4.44
40–44	0.50	0.20	1.25	1.91	0.54	6.75	1.25	0.40	3.89
45–49	0.51	0.17	1.54	3.75	0.70	20.09	1.20	0.29	5.02

*Number of children*									
0–1 (ref)	**----**	**----**	**----**	**----**	**----**	**----**	**----**	**----**	**----**
2–5	1.54	0.89	2.68	0.66	0.33	1.31	**0.62**	**0.40**	**0.97**
6 +	1.09	0.51	2.36	0.71	0.20	2.47	0.67	0.33	1.35

*Education*									
None (ref)	**----**	**----**	**----**	**----**	**----**	**----**	**----**	**----**	**----**
Primary	0.96	0.62	1.48	1.76	0.39	7.87	1.44	0.68	3.05
Secondary	0.86	0.52	1.43	1.58	0.42	5.99	1.90	0.79	4.54
Tertiary	0.40	0.14	1.12	0.39	0.02	6.69	0.82	0.26	2.61

*Marital status*									
Married (ref)	**----**	**----**	**----**	**----**	**----**	**----**	**----**	**----**	**----**
Divorced/widowed	0.69	0.35	1.37	1.39	0.75	2.58	0.74	0.41	1.34
Never married	**0.40**	**0.20**	**0.80**	**0.31**	**0.10**	**0.93**	**0.24**	**0.08**	**0.71**

*Wealth quintile*									
1 (lowest, ref)	**----**	**----**	**----**	**----**	**----**	**----**	**----**	**----**	**----**
2	1.33	0.81	2.18	1.67	0.53	5.23	0.77	0.43	1.36
3	1.27	0.72	2.23	1.75	0.57	5.34	0.75	0.34	1.65
4	1.19	0.61	2.32	2.70	0.67	10.98	0.89	0.46	1.74
5	1.48	0.51	4.24	**4.82**	**1.50**	**15.47**	0.42	0.16	1.08
Heard FP on radio	1.03	0.71	1.48	**1.79**	**1.09**	**2.94**	0.71	0.40	1.27
Saw FP on TV	1.28	0.85	1.93	0.80	0.40	1.61	1.52	0.96	2.42
Saw FP in magazine	1.14	0.68	1.93	0.61	0.29	1.28	0.70	0.44	1.10
Number of FP methods known	0.97	0.93	1.00	0.95	0.87	1.05	1.05	0.92	1.19
Urban residence	0.56	0.29	1.07	**----**	**----**	**----**	0.92	0.33	2.56

*Survey year*									
2016 (ref for Burkina, DRC)	**----**	**----**	**----**	**----**	**----**	**----**			
2017 (ref for Uganda)	**1.89**	**1.04**	**3.42**	0.95	0.44	2.07	**----**	**----**	**----**
2018	**2.13**	**1.12**	**4.08**	1.89	0.76	4.72	**2.20**	**1.15**	**4.22**
2019	**2.59**	**1.33**	**5.01**						

Instances where the ORs in 95% CIs do not cross 1 are indicated in bold font.

Next, we compared users of DMPA-SC with women not using any modern method of contraception (Table 3). Women aged 20–24 in DRC (AOR 5.91, 95% CI 2.34–14.92) and 25–29 in Uganda (AOR 3.66, 95% CI 1.39–9.62) had significantly greater odds of using DMPA-SC than women aged 15–19; women aged 45–49 had lower odds of using SC than 15–19-year-old women in Burkina Faso (AOR 0.22, 95% CI 0.08–0.64). Women with more children were more likely to be using DMPA-SC in Burkina Faso. Currently unmarried women were less likely to be using DMPA-SC than married women in Burkina Faso and Uganda. As with previous results, women in the highest wealth quintile as well as women who had heard an FP advertisement on the radio were more likely to be using DMPA-SC in DRC.

**Table 3:** Weighted logistic regression results for differences in characteristics between DMPA-SC and nonusers of modern contraception, Burkina Faso, DRC and Uganda: PMA2020, 2016–2019

*N* =	Burkina Faso			DRC			Uganda		
	AOR	95% CI		AOR	95% CI		AOR	95% CI	
	9975			10077			6206		
*Age category*									
15–19 (ref)	**----**	**----**	**----**	**----**	**----**	**----**	**----**	**----**	**----**
20–24	1.73	0.93	3.23	**5.91**	**2.34**	**14.92**	2.19	0.90	5.32
25–29	1.45	0.74	2.85	3.03	0.92	9.94	**3.66**	**1.39**	**9.62**
30–34	1.13	0.51	2.48	2.29	0.59	8.82	2.37	0.90	6.24
35–39	0.90	0.37	2.16	1.93	0.54	6.85	2.76	0.97	7.89
40–44	0.57	0.28	1.19	1.57	0.45	5.47	1.30	0.39	4.28
45–49	**0.22**	**0.08**	**0.64**	1.52	0.31	7.39	0.90	0.21	3.80

*Number of children*									
0–1 (ref)	**----**	**----**	**----**	**----**	**----**	**----**	**----**	**----**	**----**
2–5	**2.69**	**1.69**	**4.28**	1.28	0.74	2.20	1.20	0.83	1.74
6 +	**2.82**	**1.50**	**5.32**	2.07	0.76	5.60	1.46	0.87	2.45

*Education*									
None (ref)	**----**	**----**	**----**	**----**	**----**	**----**	**----**	**----**	**----**
Primary	**1.64**	**1.03**	**2.62**	1.87	0.39	8.92	2.08	0.97	4.43
Secondary	**1.79**	**1.03**	**3.14**	2.00	0.41	9.72	**3.23**	**1.33**	**7.84**
Tertiary	1.03	0.37	2.90	0.49	0.02	9.67	1.52	0.50	4.59

*Marital status*									
Married (ref)	**----**	**----**	**----**	**----**	**----**	**----**	**----**	**----**	**----**
Divorced/widowed	0.52	0.28	0.96	1.52	0.85	2.72	**0.50**	**0.25**	**0.99**
Never married	0.19	0.09	0.44	0.45	0.17	1.25	**0.18**	**0.05**	**0.62**

*Wealth quintile*									
1 (lowest, ref)	**----**	**----**	**----**	**----**	**----**	**----**	**----**	**----**	**----**
2	1.59	0.99	2.56	2.23	0.64	7.70	1.09	0.59	1.99
3	1.40	0.83	2.35	2.66	0.84	8.42	1.47	0.63	3.46
4	1.46	0.79	2.70	4.11	0.94	18.07	1.85	0.92	3.73
5	2.02	0.63	6.49	**6.94**	**2.07**	**23.24**	0.91	0.31	2.67
Heard FP on radio	1.35	0.93	1.94	**2.18**	**1.26**	**3.79**	0.66	0.38	1.13
Saw FP on TV	1.40	0.94	2.09	0.91	0.38	2.15	**1.70**	**1.04**	**2.78**
Saw FP in magazine	1.28	0.72	2.27	0.81	0.37	1.75	0.74	0.46	1.21
Number of FP methods known	0.99	0.96	1.03	1.02	0.90	1.15	1.12	0.99	1.26
Urban residence	0.78	0.41	1.49	**----**	**----**	**----**	0.92	0.32	2.65

*Survey year*									
2016 (ref for Burkina, DRC)	**----**	**----**	**----**	**----**	**----**	**----**			
2017 (ref for Uganda)	1.72	0.95	3.11	1.07	0.46	2.46	**----**	**----**	**----**
2018	**2.73**	**1.48**	**5.01**	**2.70**	**1.06**	**6.85**	**2.37**	**1.27**	**4.42**
2019	**3.39**	**1.87**	**6.12**						

Instances where the ORs in 95% CIs do not cross 1 are indicated in bold font.

## 4 Discussion

Our results show that DMPA-SC was growing rapidly in Burkina Faso, DRC and Uganda, both among all women and married women. These increases were monotonic and substantial over a relatively short period of time. These trends are consistent with rapid increases in DMPA-SC availability at service delivery points in Burkina Faso [7].

For many users of DMPA-SC, this was their first contraceptive method. Most DMPA-SC users in Burkina Faso and Uganda and more than one third of users in DRC reported as being first-time users of contraception. This number exceeds estimates from the four-country pilot test of DMPA-SC, which reported that 29% of all DMPA-SC doses administered were to new contraceptive users [5].

Many of the characteristics associated with use of DMPA-SC varied across countries. Women living in wealthier households and those who heard an FP advertisement on the radio were more likely to use DMPA-SC in DRC; this was not the case in other countries. The relationship between age and DMPA-SC varied, with women aged 20 to 24 more likely in DRC, compared to 25 to 29 in Uganda, and no relationship between age and use in Burkina Faso. Some of these differences may be explained by family planning programs in each country; DRC has an FP radio campaign targeted at youth aged 18–24 [14]. Our results here are consistent with the target audiences of this campaign.

However, some results were consistent across settings. It is important to note that we found no evidence that younger women (aged 15–19) were more likely to use DMPA-SC in any geography; in fact, we found limited evidence of a variation in DMPA-SC use by age overall. Also, never-married women were significantly less likely to use DMPA-SC compared to other modern methods. Level of education was positively associated with DMPA-SC use (compared to nonuse) in Burkina Faso and Uganda.

Overall, we find that DMPA-SC is an increasingly popular method, which supports results from pilot tests that demonstrated the appeal of DMPA-SC, particularly among first-time users of contraception [5]. This continued popularity may encourage further investment in expanding access to DMPA-SC in order to achieve their family planning goals. The fact that patterns of use differed across settings is notable but not surprising given the variation across countries in characteristics of the pilot tests in terms of scope and service delivery channel (as described in Stout et al., 2018 [5]).

There are some limitations in this research. Although use of DMPA-SC is increasing, it is still at relatively low levels in these countries, which lead to small sample sizes, large CIs and limited ability to detect statistical interactions in some cases. Some measures that might be related to DMPA-SC use are not available in the PMA2020 data, such as access to DMPA-SC, knowledge of DMPA-SC specifically (as opposed to knowledge of injectables in general) and urban residence in DRC. Given the multiple ways in which DMPA-SC can be distributed within a community, it would be useful to know how DMPA-SC is administered to the woman: is it self-administered or administered by a trained provider at a fixed facility, or by a community worker? Some research suggests that method continuation may vary by how DMPA was administered [[15], [16], [17]], although other research shows no difference [[18], [19], [20]]. It would also be useful to know the total cost of DMPA-SC for the client, as this could be an important factor for scale up and patterns of use. We also note that, because we do not examine differences between DMPA-SC and DMPA-IM, we cannot tell whether differences between DMPA-SC and all other modern methods are specific to DMPA-SC users or may apply to all users of injectables. Finally, just two data points for Uganda may not be sufficient to constitute a “trend” per se.

Our next steps follow from the above. An important related topic is method switching: does DMPA-SC use come at the cost of DMPA-IM, or are DMPA-SC users switching from other methods, and which ones? We do not thoroughly examine this question here due to space constraints, but our results showed that the majority of DMPA-SC users did not switch from another method in Burkina Faso and Uganda, which are consistent with other research showing few women switching from DMPA-IM to DMPA-SC [5]. This has implications for the contribution of DMPA-SC use to the prevalence of modern contraceptive use, as new users can increase the MCPR in contrast to women switching methods. We intend to investigate this further in the future. In addition, we do not know whether and how DMPA-SC users are different from other modern methods individually; we intend to examine this as well. We also intend to expand the geographic scope of this research as more countries expand distribution of DMPA-SC, and we plan to incorporate a question on who administered the DMPA-SC injection in countries where self-injection is being supported and promoted. Given the rapidly increasing use of DMPA-SC, we expect research of this nature to be increasingly relevant for FP programs and policies in SSA in the coming years.
